# Individual and social defenses in *Apis mellifera*: a playground to fight against synergistic stressor interactions

**DOI:** 10.3389/fphys.2023.1172859

**Published:** 2023-07-07

**Authors:** Joy Gaubert, Pierre Giovenazzo, Nicolas Derome

**Affiliations:** ^1^ Laboratoire Derome, Département de Biologie, Institut de Biologie Intégrative et des Systèmes, Université Laval, Québec, QC, Canada; ^2^ Laboratoire Giovenazzo, Département de Biologie, Université Laval, Québec, QC, Canada

**Keywords:** honeybee, synergy, stressors, detoxification, social defenses

## Abstract

The honeybee is an important species for the agri-food and pharmaceutical industries through bee products and crop pollination services. However, honeybee health is a major concern, because beekeepers in many countries are experiencing significant colony losses. This phenomenon has been linked to the exposure of bees to multiple stresses in their environment. Indeed, several biotic and abiotic stressors interact with bees in a synergistic or antagonistic way. Synergistic stressors often act through a disruption of their defense systems (immune response or detoxification). Antagonistic interactions are most often caused by interactions between biotic stressors or disruptive activation of bee defenses. Honeybees have developed behavioral defense strategies and produce antimicrobial compounds to prevent exposure to various pathogens and chemicals. Expanding our knowledge about these processes could be used to develop strategies to shield bees from exposure. This review aims to describe current knowledge about the exposure of honeybees to multiple stresses and the defense mechanisms they have developed to protect themselves. The effect of multi-stress exposure is mainly due to a disruption of the immune response, detoxification, or an excessive defense response by the bee itself. In addition, bees have developed defenses against stressors, some behavioral, others involving the production of antimicrobials, or exploiting beneficial external factors.

## 1 Introduction: domestic bees and their importance on our modern society

Although it is difficult to accurately date the beginnings of bee domestication, the earliest evidence of beekeeping practices appears to date back to ancient Egypt in the form of funerary paintings (2,400, 1,450 and 600 BC) (Crane, 2004). To attract bees, early beekeepers simply placed collected combs in boxes or cylinders made of natural materials (Weber, 2013). Similarly, *Apis mellifera*, the only native European honey bee, was domesticated during Roman times where beekeepers influenced their swarming to take them to desired locations (Crane, 2004; Weber, 2013). It was not until the 1,600 s that humans introduced this species to North America and most of the habitable areas of the globe (VanEngelsdorp and Meixner, 2010). Currently, *A. mellifera* is present all over the world (except Antarctica) thanks to human management (Geslin et al., 2017).

### 1.1 Economic and ecological weight of the domestic bee

Although the honeybee *Apis* spp. is mostly known for its production of honey, it would be reductive to link its economical weight only to the honey market. Firstly, bees also generate many other commercial hive products, such as beeswax, propolis, royal jelly and venom. Honey production alone is valued at USD 118 million annually in Canada (Agriculture et Agroalimentaire Canada, 2017), USD 718 million annually in the US (Matthiews et al., 2018), USD 186 million annually in France (FranceAgriMer, 2020) and USD 39 million annually in Australia (Batt and Liu, 2012) for examples. Furthermore, all bee products have antimicrobial properties and specific characteristics with great potential for the formulation of new drugs. For example, honey and propolis have been identified as effective for treating the *Herpes simplex* virus (Rocha et al., 2022), while propolis and royal jelly have shown therapeutic potential for treating cancer and neural diseases (Badria et al., 2017; Ali and Kunugi, 2020). Bee venom is currently being investigated for its potential application against various diseases (Son et al., 2007; Khalil et al., 2021). Thus, the honeybee has great potential for the pharmaceutical market in the future, especially because bee products are complex substances that are therefore difficult to synthesize. On this aspect, some breeds such as the European honeybee have been extensively studied but research on products of other bee breeds is growing. For example, stingless bee products are increasingly studied as they seem increasingly promising (Zulkhairi Amin et al., 2018; Al-Hatamleh et al., 2020; Mustafa et al., 2020). Nevertheless, the most critical economic contribution of the honeybee lies in its ability to pollinate various important crops essential for human nutrition. *Apis mellifera* is indeed the most important commercially available crop pollinator in the world (Garantonakis et al., 2016). At least 35% of the world’s food production depends on insect pollination and honeybees are the most manageable pollinator for this purpose (Klein et al., 2007). In Europe, 10% of the total economy of food production involves pollinators (Gallai et al., 2009). Honey bees are known to pollinate over 100 commercial plant species in North America (Hung et al., 2018; Hristov et al., 2020a; Khalifa et al., 2021). Moving and renting beehives is now a common practice as in Québec (Canada) for blueberry crops, or in California (United States) for almond orchards (DeGrandi-Hoffman et al., 2019). Bees’ pollination contributes USD 3.07 billion per year to American agriculture and more than 200 billion worldwide (Gallai et al., 2009; Khalifa et al., 2021). In 2020, the estimated global value of crop pollination services ranged from USD 195 billion to USD 387 billion, an increase that could be explained by the rising production costs of pollinator-dependent crops (Porto et al., 2020). They also improve crop quality (Giannini et al., 2015) as observed with coconuts in India (Meléndez-Ramírez et al., 2004) or with sesame in Burkina Faso (Stein et al., 2017). Bee pollination can be said to improve the profitability and productivity of many crops essential for human nutrition such as fruits, vegetables, seeds, nuts or coffee, for example, (Khalifa et al., 2021). The presence of beneficial insects in crops is known to reduce the abundance of pests, making them even more valuable (Stern et al., 1959). Finally, by pollinating wild species, bees provide ecological services, defined as “functions provided by nature that sustain and improve human wellbeing” (Daily, 1997). Such services include carbon sequestration, water purification or biodiversity preservation among others (see Hristov et al., 2020b). These pollination services are essential to ecosystems and thus to humans, but they would be difficult to quantify in monetary terms, which does not make them any less precious.

### 1.2 Role of the domestic bee in pollinator and ecosystem conservation

Like other pollinators such as wild bees, honeybees maintain functional ecological communities in nature by providing pollination services. Indeed, around 80% of wild plants require insects for their reproduction. Nevertheless, only 5% of plant species appear to be strictly visited by *A. mellifera* (Hung et al., 2018), perhaps explaining why their importance is sometimes minimized. The main argument from that perspective is that the honeybee pollination rate is negligible in comparison to that of other species. This is indeed true in some respects, as honeybees are imported and therefore not specialists of local plants (Ollerton et al., 2012). Nevertheless, their large population size could compensate for this low efficiency, as could the fact that they are also generalists, allowing them to pollinate a wider range of species, especially species neglected by wild pollinators (Aebi et al., 2012). Their numbers, communication patterns and social organization strengthen their contribution. Indeed, thanks to their informative dances, foragers can precisely indicate the location of a floral resource, inform others not to go there in case of overcrowded sites, and ask other bees to help them unload their harvest (Hrncir et al., 2011; Broccard-Bell, 2021). In this way, honeybees avoid wasting time searching for resources, making them very efficient pollinators.

In addition, studying the honeybee can contribute to the conservation of other pollinators. Indeed, both honeybees and wild pollinators are currently facing many threats and suffering huge losses (Winfree, 2010). To this end, the honeybee could serve as a bioindicator of environmental contamination with relevant data having much wider implications. Thus, the study of honeybees and their products could allow scientists to detect areas where other pollinators may be at risk, whether because they are heavily contaminated by pesticides or colonized by invasive species or because their climate has changed, for example, (Celli and Maccagnani, 2003; Bargańska et al., 2016). Once such areas have been detected, it would be easier to implement targeted conservation measures to save all pollinators or reduce risks. This strategy might only work at the individual level, given the differences in wild pollinator and honeybee lifestyles (Wood et al., 2020). In addition, some studies show negative correlations between honey bee colony density and wild pollinator activity, particularly in urban areas (Wojcik et al., 2018; Ropars et al., 2019). Concern is also growing about the possible transmission of pathogens from honey bee colonies to wild pollinators (Graystock et al., 2013). These should be considered when placing honey bee colonies. Nevertheless, studies at this scale could be of great interest. The honeybee could indeed be a model for genetic studies, especially since its genome is now fully sequenced (Honeybee Genome Sequencing Consortium, 2006).

From a more political point of view, bees are a very famous species. The slogan “Save the bees” has now become a universal slogan and helps spread the message of the importance of biodiversity and the threats to it to the widest audience. Topics favored by public opinion attract more funding and visibility and allow related projects to receive funding as well. Therefore, studying honeybees and trying to save them is a way to support doing the same for other pollinators, making them more familiar to people and potential funders.

### 1.3 Bee decline

Given the importance of honeybees from an economic and ecological standpoint, it is not surprising that humans have long been multiplying the number of honeybee colonies (Aizen and Harder, 2009), to meet the demand for pollination services. More recently, the health of these colonies is increasingly alarming. Indeed, beekeepers are experiencing increasing numbers of colony losses over the years, often related to high winter mortality, primarily in North America and Europe (Gray et al., 2020; Canadian Association of Professional Apiculturists, 2022). Given the global scale of the phenomenon, it is very unlikely to result from a single specific stressor. Although it is difficult to fully understand the causes, scientists agree that the combination of various stressors and their interactions are leading to bee decline. By the term “stressors,” we mean any element, whether biotic (parasites, viruses, predators) or abiotic (pesticides, nutrient stress, temperature), that disturbs the proper internal functioning of the bee or the colony. Because of their social nature, lifestyle, and foraging behavior, honey bees are exposed to a multitude of these stressors. Indeed, the proximity of thousands of genetically close individuals within a hive facilitates the development and dispersal of pathogens (Fries and Camazine, 2001). It is their foraging behavior that brings them into contact with anthropogenic stressors, as well as beekeepers’ treatments (Fulton et al., 2019; Xiao et al., 2022), which can also be detrimental in certain circumstances for honey bees (e.g., intestinal dysbiosis, brood toxicity) (Pettis et al., 2004; Thompson et al., 2005; Daisley et al., 2020; Jia et al., 2022). In the context of deteriorating bee health, the spread of new parasites, increasing exposure to pesticides and hive treatments, beekeeping practices, diminishing floral resources, and declining queen quality could be the main factors responsible for this tragedy (Smith et al., 2013; Hristov et al., 2020a). In their study published in 2019, Neov et al. (2019) identified other factors they consider to be responsible for bee colony mortality. The varroa mite and the community of viruses it transmits to bee brood are identified as the primary cause, followed by landscape change and reduced access to food resources, beekeeping practices (including excessive transport in the US), and intensive farming with its spread of pesticides. They also describe how climate change has always impacted bee populations in the past and how they have always managed to recover. This supports the theory that colony mortality is not associated with a single factor, but rather the simultaneous accumulation of multiple stressors. Herein lies the complexity of the decline of bee health. The exposure of bees to stressors of the same type (i.e., several different pesticides) can potentially undermine their defense systems (Berenbaum and Johnson, 2015). In the event of a combination of different types of stressors (i.e., biotic and abiotic), several types of defenses may be activated at the same time, such as the immune system and the detoxification (Mao et al., 2013). This can lead the bee’s body to prioritize one type of defense, leaving the field open to the stressors of the other type. It can also trigger a general weakening of the individual, as its reserves of resources and energy are drained in the fight against stressors (Dussaubat, 2012; Buchon et al., 2014). If food resources are scarce at the time, the bee’s exposure to all these stressors at once can even be fatal (DeGrandi-Hoffman and Chen, 2015). Some stressors can even impair the immune system or the detoxification (Di Prisco et al., 2013). In addition, some stressors can interact with each other, generating additive, synergistic or antagonistic effects (O'Neal et al., 2017; Raymann et al., 2018; Tosi and Nieh, 2019). It is therefore essential to understand the underlying mechanisms of these interactions between stressors, their individual effects, as well as their overall impacts on bee health and defense systems, if one is to have any chance of halting this decline.

### 1.4 Goals and challenges of this review

This review aims to gather and provide information on the exposure of honeybees to multiple stressors, as well as their defense mechanisms. The first part inventories interactions and synergistic/antagonistic effects between the most common stressors of honeybees and describes (the processes) involved. This knowledge will help to obtain an overview of both exposure to multiple stresses and their consequences. Furthermore, such knowledge could be used to predict and perhaps even avoid the impacts of these stressors on bee health. In order to consider solutions to fight or mitigate the effects of these multiple stresses, defense strategies and the beneficial external factors that help honeybees to resist and maintain their health will be presented and discussed in a second part.

## 2 Exposure of bees to multiple stresses

Since the Late Cretaceous, about 100 million years ago [Michener and Grimaldi (1988)], honeybees and other pollinators have been exposed to certain stressors, such as predators, pathogens, or suboptimal environmental conditions. Despite this, honeybees have survived and evolved. Unfortunately, urbanization, globalization and the increase of modern agriculture have introduced a wide range of new stressors for honeybee colonies. In addition, their specific lifestyle may increase their exposure to these stressors. By living in communities of many individuals gathered in enclosed, temperate locations, honeybees experience a different degree of exposure to stress than other pollinators. Indeed, it seems relatively easy for pathogens to spread from one individual to another (horizontal transmission) when these individuals interact very closely (Fries and Camazine, 2001). Furthermore, all of these individuals have very similar genetics and the same defense systems because they are the progeny of the same queen.

**FIGURE 1:**
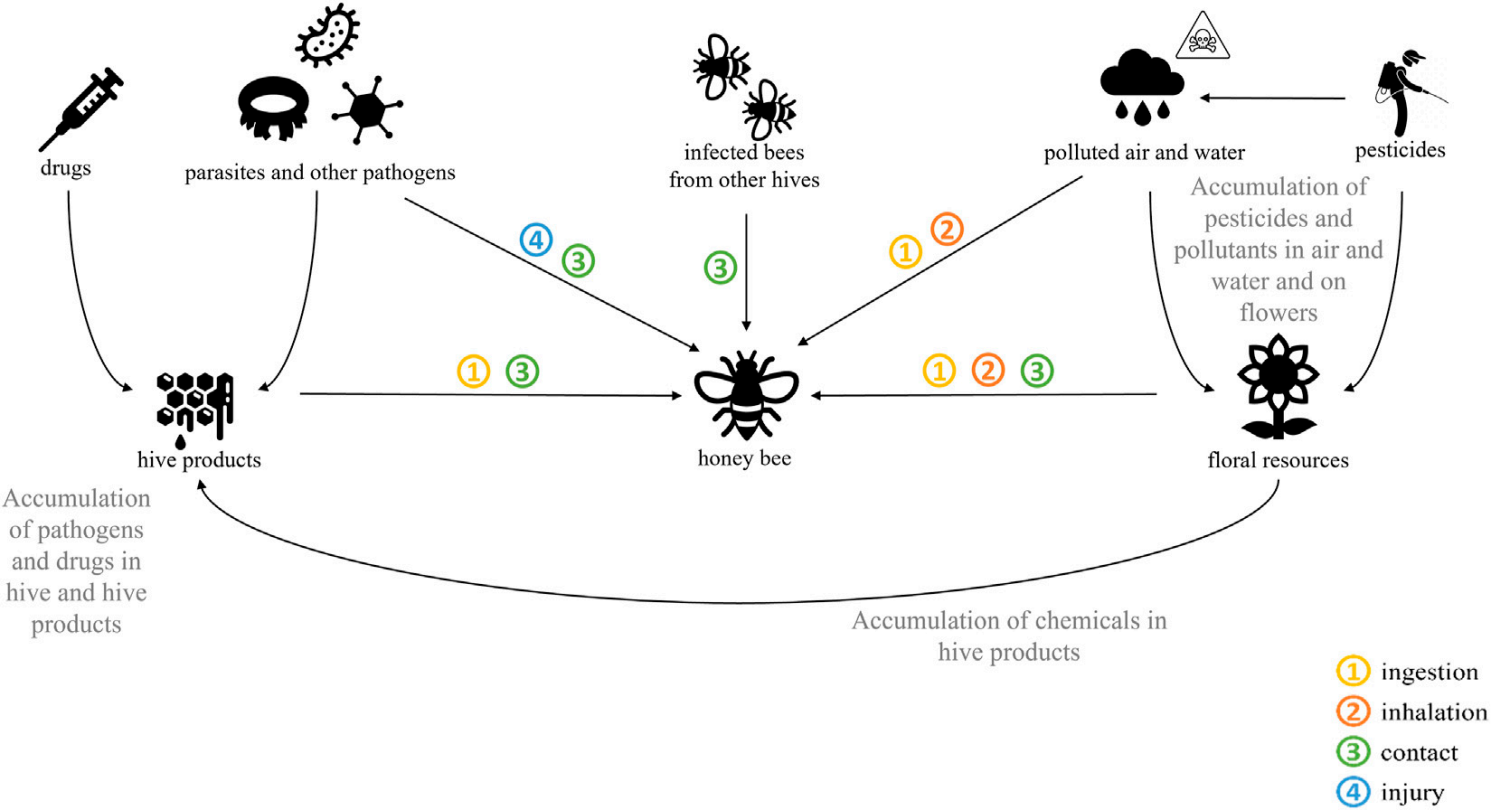
Main routes of contamination of honey bees by stressors.

A second major source of bee exposure to stress is external, through exposure to various drugs. Beekeepers regularly use chemical molecules, such as antibiotics or acaricides, to fight against pathogens. These chemicals can then remain in the hive and accumulate. As bees gather pollen and nectar, they may simultaneously be exposed to a considerable variety of agro-chemical molecules, such as pesticides or herbicides and insecticides. Chemicals and pesticides can then accumulate in hive products, including wax, bee bread, and pollen (Daniele et al., 2018; Fulton et al., 2019; Xiao et al., 2022). These molecules can lead to lethal damage, but they also induce a large range of sublethal effects that weaken the fitness of impacted colonies (such chronic exposure is well described in the review by Chmiel et al., 2020). Bee products can be contaminated by wind-borne particles or polluted water. Contamination routes are summarized in Figure 1. Thus, bees can be exposed to biotic and abiotic stressors through different routes of contamination. The main route of contamination is consumption and absorption of the stressor, but bees can be contaminated topically or orally by sprayed pesticides, for example. The most dangerous route is often the oral route: the bees will swallow the contaminant by eating pollen, nectar, or bee bread or by feeding new bees by trophallaxis. This is the case for the intestinal parasite *Vairimorpha (Nosema) ceranae* and various pesticides whose LD_50_s (the dose of the stressor at which 50% of the population dies) are lower and therefore more toxic by the oral route than by contact (Laurino et al., 2011; Blacquiere et al., 2012; Zhu et al., 2014). In addition to simple contact between contaminated and healthy bees, some stressors can be spread by parasites. For example, the varroa mite feeds on the hemolymph of bees and on the larval fat body, inflicting deep wounds and infecting the host with a wide range of bee viruses such as black queen virus (BQCV) and deformed wing virus (DWV) (Dussaubat, 2012; Mondet et al., 2014). Finally, pathogens such as viruses can be passed among bees during the reproductive process, from drones to the queen or from the queen to her brood, in a vertical transmission route (Ravoet et al., 2015; Prodělalová et al., 2019).

## 3 Interactions between stressors

### 3.1 Global increase of adverse effects with multiple exposures

Because bees are often exposed to many different stressors and most often to several stressors simultaneously, recent studies more frequently include cross-exposure trials (Tosi and Nieh, 2019; Paris et al., 2020; Lupi et al., 2021; Balbuena et al., 2022). In the future, such studies could allow us to adapt or develop new strategies to increase bee resiliency and resistance to multiple coexistent stressors. In this section, some examples of exposure to multiple stressors will be described and discussed in order to explain their impacts on bee health and productivity.

An example of synergy is double parasite exposure to *Varroa destructor* and *Vairimorpha ceranae*. In Little et al. (2015), found that the intensity of *V. ceranae* infestation was positively correlated with that of *V. destructor* and *vice versa*. Another example of synergy involving *V. ceranae* was documented by Schwarz and Evans (2013). After comparing the defenses developed by the bee against exposure to this parasite and the arthropod-specific trypanosome *Crithidia mellificae* alone, they observed that infected bees secreted fewer antimicrobial peptides and that infestation with both stressors at the same time decreased bee cellular immunity.

Pathogens can act in synergy with abiotic stressors too. Upon exposure to pesticides (especially fungicides and acaricides), honeybees are twice as likely to be infested with *V. ceranae* (Wu et al., 2012; Pettis et al., 2013). Similarly, bees become more susceptible to viruses when treated with certain acaricides or pesticides. Some of these products, such as amitraz and clothianidin, promote virus replication (Di Prisco et al., 2013; O'Neal et al., 2017). Another study showed that pesticides like clothianidin or fluvalinate reduce survival of bees infested with *Peanibacillus larvae*, the pathogen of American foulbrood (López et al., 2017).

Pesticides can even act in synergy with each other. Indeed, some pesticides applied in the field interact with fungicide treatments used in the hive, which is among the worst-case scenarios as it occurs at field concentrations (Thompson et al., 2014). It occurs specifically with molecules called sterol biosynthesis inhibitors (SBI). Taken individually, these fungicides have a relatively low acute impact on bees, unlike insecticides. Nevertheless, they act synergistically with pesticides and become toxic within the insect body (Raimets et al., 2018; Iverson et al., 2019). Another example of pesticide interaction is the case of flupyradifurone, whose LD_50_ decreases 6-fold when interacting with the fungicide tebuconazole (Tosi and Nieh, 2019).

The high winter loss of honeybees observed today in the United States, Canada, and Europe may occur because of synergistic phenomena between stressors. In Desai and Curry (2016), published a study describing “the effects of overwintering environment and parasite-pathogen interactions on honeybee colony loss.” Several colonies were kept outdoors with insulating blankets during a Canadian winter, while others were kept indoors at constant low temperatures. Parasite and viral loads as well as colony survival rates were measured. They showed the critical role of beekeeping practices, as colonies overwintered outdoors had a higher survival rate than colonies overwintered indoors with the same pathogen loads. This may indicate that a constant low temperature weakens bees less than variable winter conditions during winter, whereas variable temperatures could increase the effects of parasites. Similarly, they observed an increased abundance of *V. ceranae* in colonies overwintered outdoors and a decreased abundance in colonies overwintered indoors. Most of these colonies had been treated with fumagillin previously, so these observations could be the result of increased replication of *V. ceranae* spores in winter or reduced efficacy of the treatment at low temperatures. Indeed, the properties of a chemical can vary with temperature and several pesticides were found to be more toxic at low temperatures or in the presence of other context-dependent factors (Henry et al., 2014; Saleem et al., 2020).

Other variables intrinsic to bee biology may also be important in these interactions. A given caste of bee (e.g., nurse or forager) may suffer more from the presence of a stressor than others, For example, foragers were shown to be the most infected by *V. ceranae* in the colony (Higes et al., 2008; Martín-Hernández et al., 2018). In addition, this parasite seems to exert a sex-related effect, since drones show higher mortality when contaminated. Infected drones even facilitate the spread of the parasite compared to contaminated workers (Roberts and Hughes, 2015; Martín-Hernández et al., 2018).

### 3.2 Main modes of action related to stressors interactions

The effects resulting from exposure to multiple stresses on bee health can be cumulative, but also synergistic (deleterious effects outweighing cumulative effects or appearance of new deleterious effects) or antagonistic (the presence of one stressor reducing the deleterious impact of a second). This depends on the stressor, its mode of action, the contamination route, the environment, and bee health and defense systems.

#### 3.2.1 Individual immune response in the honeybee

There are similarities between the innate immune responses of insects and vertebrates, which involve cascading reactions that trigger mechanisms such as phagocytosis, enzymatic degradation, and secretion of antimicrobial peptides (Hoffmann, 2003). Each bee also has an individual immunity including cellular and humoral responses (developed in the review of Larsen et al., 2019). Several signaling pathways have been identified in insects and especially in bees, *Drosophila*, and mosquitoes (see Evans et al., 2006 for detailed description). The first is the Toll pathway, which functions through transmembrane signaling proteins called Toll-like receptors. These proteins play a role in immunity and development. When a cytokine molecule binds to this receptor, it forms a complex that will degrade an inhibitor of the nuclear factor κB (NF-κB) that is then translocated into the nucleus. NF-κB is a gene transcription factor of the immune system that is also involved in many fundamental processes (Evans et al., 2006; Hayden and Ghosh, 2008). Its translocation into the nucleus allows the activation of several effectors such as antimicrobial peptides, phenoloxidase and lysosomes. The immune deficiency (IMD) pathway plays a more specific role in fighting against bacteria (especially Gram + bacteria) but can also be triggered in the presence of certain fungi. It induces the transcription of major antimicrobial peptides (AMP) via the NF-κB transcription factor Relish. These defense systems occur primarily in the gut. The bee must therefore maintain a balance between the pathogenic and commensal bacteria in its gut microbiota. When the PGN (bacterial cell wall compound) of a bacterium is recognized by a peptidoglycan recognition protein (PGRP), there are two possible outcomes. In the case of sustained association with low numbers of bacteria, the intestinal master sensor PGRP-LE induces local production of amidase, which can cleave PGNs and prevent their diffusion into the hemolymph (which activates the IMD pathway). It is also a negative regulator of NF-κB that adjusts its signaling level to the nature of the bacteria. This allows a protective immune tolerance towards the microbiota. In contrast, under high loads of infectious bacteria, the PGRP-LE response switches to AMP production through the IMD pathway (via the transcription factor NF-κB Relish) to eliminate the stressor (Bosco-Drayon et al., 2012). A subsequent reduction in the number of immune genes has been observed in domestic bees compared to *Drosophila* and mosquito. This reduction occurs at each step of the immune pathways and could be related to the presence of specific social defenses that render these additional genes unnecessary (Evans et al., 2006; Larsen et al., 2019).

#### 3.2.2 Detoxification in the honeybee

It is not widely known that bees possess yet another system to fight xenobiotics. Certain foreign and non-living substances present in the organism are able to interact with host cells. They can be natural (plant toxins) or anthropogenic (such as pesticides) (Berenbaum and Johnson, 2015). The objective of detoxification is to transform lipophilic substances into hydrophilic substances so that they can be excreted easily (Berenbaum and Johnson, 2015; Chen, 2020). This process consists of three phases. The first is called functionalization and aims to cut the lipophilic interaction through enzymatic alteration. Enzymes used in this phase belong to the cytochrome P450 superfamily or are carboxylesterases (Berenbaum and Johnson, 2015; Hilliou et al., 2021). Bees use them against flavonoids in their food but also against mycotoxins, pesticides and acaricides (Hýbl et al., 2021). The second phase is the conjugation of the product of the first phase to a glutathione thanks to a glutathione-S-transferase so that it is solubilized and transportable. In the third phase of detoxification, the final metabolite is transported out of the cell until its excretion. This is done through ABC (ATP-binding cassette) proteins such as multidrug-associated proteins (Berenbaum and Johnson, 2015). This three-phase process takes place in the midgut, where detoxification enzymes are present (Glavan and Bozic, 2013).

Domestic bees suffer from a genetic deficit in enzymes that operate during the three phases compared to other insects. They have fewer variants of the enzymes involved. This does not render the bee more sensitive to pesticides since the LD_50_s associated with these pesticides are comparable to those of other insects (Hardstone and Scott, 2010). Nonetheless, this deficiency could partially explain the sensitivity of bees to synergists. Reviews by Berenbaum and Johnson (2015) and Gong and Diao (2017) provide a detailed description of this phenomenon.

#### 3.2.3 Modes of action related to synergic interactions

**FIGURE 2:**
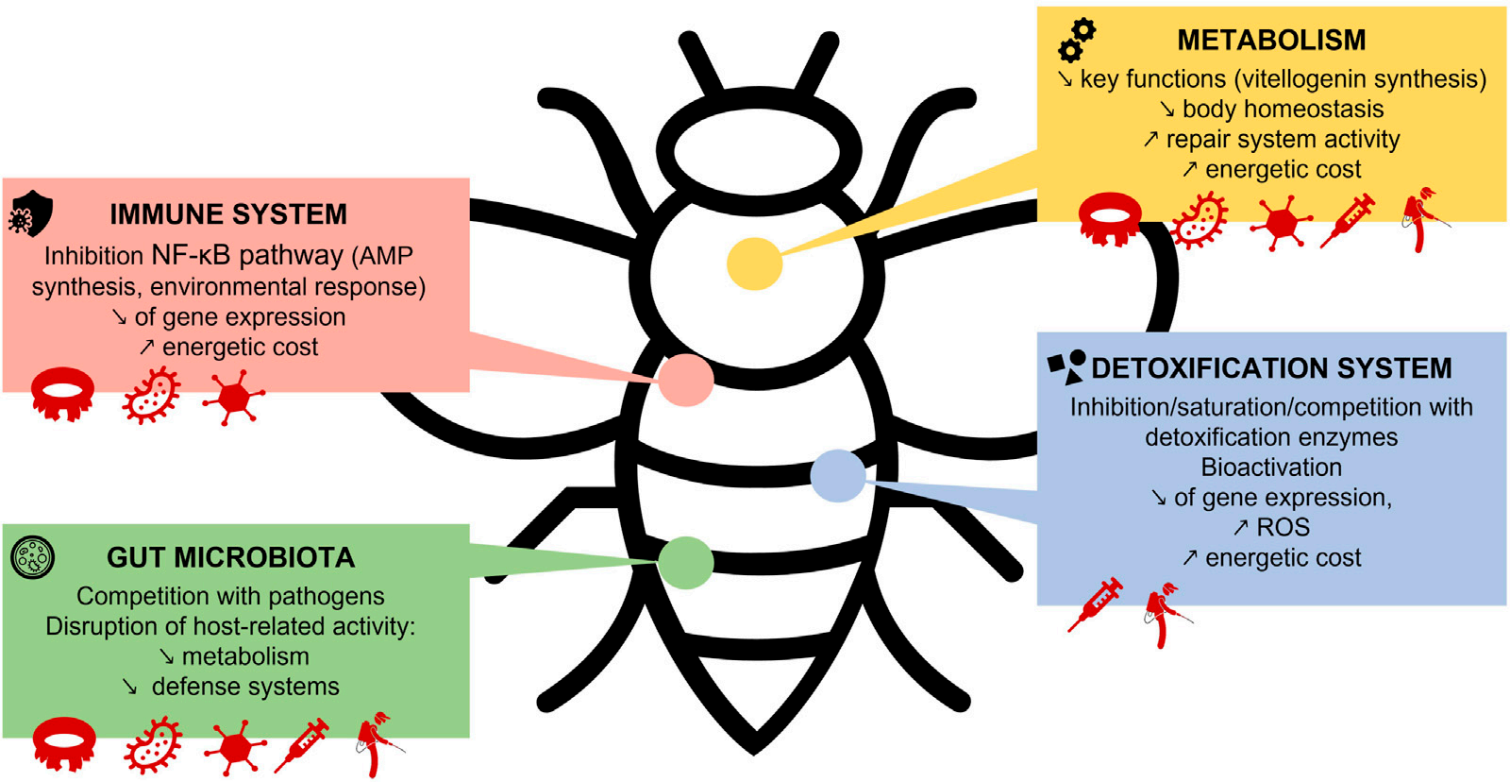
Impacts of exposure to multiple stressors on honey bee defense systems and metabolism.

The way in which synergistic interactions between stressors can be so detrimental to bees is very complex and still little understood. Indeed, each context has its own explanations depending on mechanisms that are often unknown. Nevertheless, studies are increasingly addressing this subject, and have identified some of the modes of action that lead to such synergistic effects. The impacts on honeybee defense and metabolic systems of exposure to multiple stressors are illustrated in Figure 2.

First, the detoxification process exacts an energetic cost on the organism (Derecka et al., 2013). Expressing the genes and encoding the proteins to detoxify and transport the toxin out of the body consumes a great deal of energy and resources. For this reason, toxicity curves are often higher for high doses but still high for low doses, as observed by El Khoury et al. (2021) where bees were exposed to clothianidin. This can be explained by the fact that below a certain threshold of xenobiotics in the organism, the detoxification process does not begin because the dose is considered too low to have an impact on the organism and the start of detoxification would cost more than the benefit for the bee. Conversely, the detoxification process is stopped at high doses in order to conserve the energy needed to maintain vital functions. In the case of other stressors, such as parasites or viruses, the body may have to “choose” between starting detoxification or the immune system response. This increases the detrimental effects of the unmanaged stressor. This is also why the presence of stressors is so deleterious in terms of energy expenditure (Derecka et al., 2013; Tosi et al., 2017).

The sensitivity of bees to xenobiotic synergy may also be explained by detoxification gene deficiency (Berenbaum and Johnson, 2015). Indeed, exhibiting multiple isoforms of detoxification enzymes may allow an organism to handle a greater diversity of chemicals. Furthermore, some isoforms may have cross activity properties, thus improving resistance against a chemical that is able to interact in a way that disarms other enzyme isoforms. Many modes of action of pesticide synergies involve the cytochrome P450 family of detoxification enzymes. For example, the adverse effects of synergy between sterol biosynthesis inhibitor (SBI) fungicides and acaricides/insecticides are likely due to inhibition of P450-mediated detoxification of pesticides by fungicides (Johnson et al., 2013). Indeed, prochloraz, which is an SBI fungicide, has been shown to inhibit P450 activity in honeybees, thereby increasing the toxicity of pyrethroid insecticides (Pilling et al., 1995; Johnson et al., 2006). Similarly, synergies between the acaricides tau-fluvalinate and coumaphos and other acaricides or pesticides could arise from competition or interference with, or saturation of, the cytochrome P450 active site due to an excess of xenobiotics in the body (Johnson et al., 2006; Berenbaum and Johnson, 2015). Both compounds are lipophilic and can accumulate in beeswax, slowly building up over time. Bees may then be continuously exposed to higher concentrations that saturate their detoxification enzymes. In that case, the normal mode of action of pesticides such as neonicotinoids has a direct impact on the health of the bee, as it remains untreated in the body.

Cytochrome P450 plays an important role not only in detoxification, but also in the synergy between antibiotics and pesticides. Antibiotics are indeed used by beekeepers to combat or prevent bacterial infections such as American foulbrood in hives (Ortiz-Alvarado et al., 2020). These molecules are designed to eliminate bacteria but do not distinguish between beneficial and deleterious bacteria. As a result, they can alter the gut microbiota and trigger dysbiosis. This disruption can have adverse consequences for host health, such as altered metabolism, decreased immunity, poor survival (Li et al., 2017; Raymann et al., 2017; Liu et al., 2020) and failure of synthesis of P450 detoxifying enzyme, as it has recently been shown that gut microbiota promotes expression of genes encoding the P450 enzyme in the midgut (Wu et al., 2020). Cross exposure to antibiotics and pesticides induces a higher mortality rate that could result from deleterious changes to the gut microbiota worsen by antibiotic, which decreases expression of detoxification enzymes. As a result, the low titers of detoxifying enzymes allow the pesticide to act inside the body, causing damage. In addition, a disrupted gut microbiota is unable to perform beneficial functions, so the host will face decreased immunity and a slower metabolism. Other pathogens can then invade, worsening the gut dysbiosis and triggering disease.

The detoxification process changes the xenobiotic chemical composition in order to expel it from the body, possibly generating new metabolites more toxic than the original compound. This is the case with imidacloprid, which yields 5-OH-imidacloprid, and thiamethoxam, which converts to clothianidin (Nauen et al., 2001; Ford and Casida, 2006; Benzidane et al., 2010). These metabolites may react with the parent molecule or act synergistically with it and create further damage. Some metabolites are particularly reactive and can break up DNA molecules, creating mutations that can lead to cancer or malformations (if the bee is still developing). The case of benzo[a]pyrene (B[a]P) illustrates this concept. This molecule belongs to the family of polycyclic aromatic hydrocarbons (PAHs) and bees can be exposed to it by breathing polluted air near highways. Once in the body, B[a]P can accumulate on lipids or be taken up by the detoxification enzymes of cytochrome P450. In either case, the first phase of detoxification leads to the formation of more toxic metabolites, a process called bioactivation. Both parent and daughter molecules can generate DNA lesions or adducts that are responsible for cell death, enzymatic alterations, and cancers (Baird et al., 2005; Moreau, 2014). Another example of such harmful molecules is the family of reactive oxygen species (ROS). Transformation of some xenobiotics can lead to the emission of ROS, but they are also directly produced by the body during its normal functioning (cellular respiration). Although ROS have antimicrobial properties, they can also react with macromolecules (DNA and proteins, for example,) and lead to cell death (Dussaubat, 2012). Many types of stressors induce ROS production, both in bees and in other organisms (Simone-Finstrom et al., 2016; Paris et al., 2017). Overproduction of ROS during detoxification can thus inflict a great deal of damage and disrupt important functions by oxidizing key enzymes, for example. This situation probably occurs with most types of disruptors and may explain some of the dangerous synergies between pesticides. Indeed, excessive immune response or detoxification can be dangerous to the host under certain circumstances. On the one hand, it comes at an energetic cost, and can lead to metabolic dysregulation, resource wastage, and inflammatory diseases in insects (Buchon et al., 2014). On the other hand, it is related to the production of those ROS (Dussaubat, 2012). This is likely to occur with multiple exposures.

Many studies show how certain pesticides inhibit bees’ immune system. This is the case with neonicotinoids. In 2013, Di Prisco and collaborators showed that clothianidin and imidacloprid increase the gene expression of leucine-rich repeat protein. This protein appears to be a natural inhibitor of NF-κB, which is a signaling component and gene transcription factor of the immune system involved in many processes such as antimicrobial peptide synthesis, coagulation, melanization, and antiviral defenses and of key importance in the response to environmental stress (Evans et al., 2006; Hayden and Ghosh, 2008). Neonicotinoids are therefore responsible for decreased immune system response in bees, which in turn promotes DWV (deformed wing virus) replication, explaining the synergy between these stressors (Di Prisco et al., 2013). Pathogens can inhibit the bee immune system too. An example is the case of the infamous varroa mite, thatlowers the host’s immune defenses, then inflicts damage by feeding directly on the bees’ hemolymph and fat bodies. It also increases the prevalence of the three strains of the DWV virus (A, B and C) in infected bees (Dainat et al., 2012; Nazzi et al., 2012). In fact, these stressors seem to exhibit a form of mutualistic interaction. The mite allows direct propagation of DWV, while the virus decreases the expression of a gene belonging to the NF-κB family that reduces the available pool of this transcription factor in the bee body (Nazzi et al., 2012). This is beneficial to varroa, because it decreases wound-triggered coagulation and melanization involving NF-κB (Hayden and Ghosh, 2008). Blocking this process leaves the wound open and allows the mite to feed properly. The decrease in the NF-κB pool implies that all stress responses dependent on this transcription factor are reduced in the infected bee, making it highly vulnerable to further environmental stresses. Varroa mites also impact key bee functions such as vitellogenin storage, which is fundamental for winter survival (Dainat et al., 2012; Martin and Brettell, 2019). The microsporidia *V. ceranae* is another pathogen that can affect bee immune response. Indeed, it downregulates certain genes that encode several serine proteases and hymenoptaecins (Aufauvre et al., 2014). Serine proteases are known to be part of the regulatory cascade reactions of the immune response. Indeed, these reactions activate the Toll and phenoloxidase pathways (Buchon et al., 2009). Hymenoptaecins are antimicrobial peptides involved in the innate immunity of insects against bacteria and fungi. The microsporidia degrade and prevent apoptosis of midgut cells as well (Kurze et al., 2018). In doing so, it impairs turnover of the midgut epithelium, which is a key organ for detoxification and immunity (Thompson et al., 2014).

#### 3.2.4 Action modes related to antagonist interactions

While lack of food increases damage from most stressors in bees, the type of food also has an impact on detoxification. In Wahl and Ulm (1983), observed that bees fed high quality pollen (polyfloral pollen or rape pollen) during the first day of life were more resistant to pesticides. This phenomenon was later confirmed by other studies, showing the importance of the quality and diversity of pollen type for pesticide and pest resistance (Di Pasquale et al., 2013; Schmehl et al., 2014). A correlation between the level of protein in the diet and the level of GST (a phase 2 detoxification enzyme) has been demonstrated in spongy moths (Lindroth et al., 1990). Amino acids in the diet could indeed be useful in producing detoxification enzymes When flavonoids are present in good quality pollen, they can also activate the detoxification process and the immune system, allowing bees to manage the stressor without latency (Johnson et al., 2012; Mao et al., 2013; Berenbaum and Johnson, 2015). One substance, p-coumaric acid, induces upregulation of several genes involved in detoxification and immune system functioning (Mao et al., 2013). A quality diet also leads to healthy gut microbiota, which can upregulate the gene expression of detoxifying enzymes (Wu et al., 2020). This is an example of the way in which antagonistic effects between stressors and diet quality can manifest and may also explain the synergy between these stressors and nutritional stress.

When stressors are parasites, they may act as antagonists within the host because they occupy the same ecological niche or consume the same resources, thus competing with the host. The antagonistic interaction between *V. ceranae* and deformed wing virus (DWV) in the midgut does not occur in other tissues, indicating that they likely compete for space, function, or resources there (Costa et al., 2011). Indeed, the parasite suppresses the resources the virus requires by destroying midgut cells and thus the cellular material that the virus needs in order to replicate (Costa et al., 2011; Doublet et al., 2014; Traynor et al., 2016). This antagonistic interaction seems to be asymmetrical, as it depends on the order of infection. If *V. ceranae* establishes before DWV, it inhibits DWV infection, but the reverse does not occur (Doublet et al., 2014; Traynor et al., 2016). Protein supplementation could increase the inhibition of DWV by *V. ceranae* but this parasite could increase DWV replication with pollen supplementation instead (Zheng et al., 2019). This underlines the complexity of these interactions and the importance of other factors like nutrition. Similarly, *Serratia marcescens*, an opportunistic pathogenic bacterium for worker bees, has antagonistic interactions with other bacteria (Raymann et al., 2018). Indeed, this bacterium exhibits strain-specific anti-competitive mechanisms against other pathogenic bacteria such as *Enterobacter cloacae*. Nevertheless, although these mechanisms do not target host eukaryotic cells, they may damage some beneficial bacteria in the microbiota and increase the damage created by this one bacterium (Murdoch et al., 2011). Another example of anti-competitive mechanisms is the fungus Aspergillus fumigatus, whose mycotoxin called Fumagillin is used to prevent and treat *V. ceranae* infection in bees (Guruceaga et al., 2019).

Another mechanism illustrating antagonistic interactions is the activation of the immune system or detoxification by a stressor. In this situation, the first stressor triggers the host’s defense system, which can be deleterious to another pathogen or even prevent its establishment. For example, the parasite *V. ceranae* inhibits immune system gene expression but not the synthesis of reactive oxygen species. This suggests that oxidative stress, rather than immunity, directly blocks the replication of DWV virus (Doublet et al., 2014). The bacterium *S. marcescens* has been shown to sometimes lead to intestinal cell purging in *Drosophila* through its pore-forming hemolysin toxin (VanHook, 2016). This purging is a defensive mechanism of the intestinal epithelium to shed pathogens in the cytoplasm that may explain some antagonist interactions between *S. marcescens* and other pathogens like *V. ceranae*.

Progress has been made in understanding the modes of action by which stressors have a synergistic *versus* antagonistic impact on bees. These interactions are extremely complex, as they are specific to each combination of stressors and depend on many external factors. Yet most studies investigate exposure to a limited number of stressors, whereas bees are exposed to a multitude. Targeting host-specific defensive molecules through bioassays could help identify the stressors to which bees are exposed, leading to the development of new strategies to predict and perhaps even counteract the consequences of multiple stress exposure in honeybee colonies. The use of technology such as QSAR could be of great help. Quantitative Structure-Activity Relationships (QSAR) are mathematical models that enable prediction of the deleterious effects of a xenobiotic based on its chemical structure. In the same way, one could imagine a program where a beekeeper would enter data on the location of their hives and obtain a detailed list of threats to the colony and solutions according to the mode of action of these stressors. This hypothetical tool would allow the beekeeper to choose the best location for their colonies and select appropriate solutions to reduce exposure, risk, and damage to the bees.

## 4 Honeybees’ defenses and beneficial environmental factors

While honeybee lifestyle is likely to favor pathogenic infestations, they have developed a “social immunity” over time. This term describes how individual behaviors can reduce the transmission and impacts of pathogens at the colony level (Simone-Finstrom and Spivak, 2010). Moreover, some natural factors in their environment may help limit bee exposure to anthropogenic stressors. In addition, honeybees have developed strategies to avoid chemical contamination. Directing funding and research toward these beneficial factors could provide support to help combat bee stressors and their synergistic effects in the future. These beneficial factors are discussed below.

### 4.1 The behavioral immunity of honey bees

Studies of bee defense systems have shown a genetic deficit in their immune system and detoxification compared to other insects (Evans et al, 2006; Berenbaum and Johnson, 2015). Bees may compensate for this genetic deficit through their social defenses and have developed an array of behaviors that help them combat stressors.

First, bees have specific strategies to protect them from their co-evolved predators, as exemplified by *Apis ceranae* in regard to the Asian hornet. “Guardians” remain at the hive entrance and surround aggressive hornets by vibrating around it until it dies of hyperthermia (Villemant, 2008). It is thus the novelty of this predator in Europe that makes it so deadly for *A. mellifera* there. Bees have also developed behavioral strategies to survive temperature variations (in winter) by forming well-organized clusters (Jarimi et al., 2020).

Bees have developed a number of complex behaviors to combat insidious stressors such as parasites, fungi or bacteria. The most famous is hygienic behavior. Workers in a colony are considered to demonstrate this characteristic when they can identify and remove infected, diseased, or dead brood (Spivak and Gilliam, 1998). This behavior prevents or reduces colony infestation by fungi or bacteria, or even varroa mites. It is a good example of social immunity, where the health of the colony takes precedence over that of the individual. Grooming is another behavior developed among European bees as a defense against parasites. It involves a bee removing the parasite from itself (autogrooming) or from its sisters (allogrooming) and is particularly effective against varroa mites (Boecking and Spivak, 1999; Invernizzi et al., 2022). Both types of behavioral defenses against parasites are used by *A. ceranae*, which co-evolved with the varroa mite. Today, researchers and beekeepers are attempting to further develop these positive behaviors by using genetic selection to accelerate the co-evolution delayed by beekeeping treatments. Indeed, these treatments, necessary to the beekeepers, prevented the natural selection process that would have allowed the selection of mite-resistant colonies over the years (Le Conte et al., 2007). Mite numbers can also be reduced by other behaviors, such as swarming, a natural process but one that is rarely allowed in apiaries (Seeley and Smith, 2015). Some honeybee subspecies are also known to imprison some of their parasites; *A. mellifera capensis* encapsulates the small hive beetle in a propolis prison (Neumann et al., 2001). The European honeybee uses propolis to encapsulate dead enemies inside the hive if they are too large to be removed, to avoid decomposition and bacterial proliferation (Burdock, 1998; Fokt et al., 2010).

Honeybees can also increase the temperature of the brood in the event of foulbrood infection, to eliminate this deadly but temperature-sensitive fungus (Starks et al., 2000). Sick, infected, or parasitized workers also tend to withdraw from the colony. Although the mechanisms of this “altruistic suicide” are not yet fully understood, affected workers tend to engage in riskier behaviors and not return to the colony, when they do not directly choose to leave (Evans et al., 2006; Rueppell et al., 2010). A sick worker remaining in the colony may be recognized by a mechanism similar to that of hygienic behavior (probably bonded to pheromones) and expelled by others (Baracchi et al., 2012). Similarly, some workers engage in corpse removal tasks to prevent bacterial proliferation and the spread of disease (Trumbo et al., 1997). All of these behavioral defenses against pathogens are well described in the review by Simone-Finstrom published in 2017.

Recently, Berenbaum and Johnson (2015) proposed that honeybees may exhibit behaviors designed to avoid contamination by natural or chemical toxins, which could compensate for their lack of detoxification genes. It has been proposed that bees may detect toxins through taste neurons in their proboscis (Kessler et al., 2015), performing a kind of selective pollination (Berenbaum and Johnson, 2015). However, Kessler and colleagues showed in 2015 that, in some cases, bees favored neonicotinoid compounds. In any case, foraging on flowers of different species allows honeybees to dilute toxins by mixing different types of pollen or nectar, thereby reducing the exposure dose (Berenbaum and Johnson, 2015). In addition, beekeepers have observed a new phenomenon of “entombed pollen.” Although this behavior is still poorly understood, it appears to be related to colony mortality and the presence of high levels of pesticides in the pollen involved (Evans et al., 2009); it may represent another means used by bees to prevent chemical exposure.

These social immunity and detoxification behaviors allow bees to decrease pathogen invasion and toxin exposure with a reduced number of dedicated genes. Bees also defend the hive by spreading antimicrobial compounds from various sources, as described below.

### 4.2 Bee collective defenses in the hive

Social bees have also developed a true collective immunity, with defensive molecules and beneficial bacteria distributed throughout the hive. To visualize this, one could imagine the colony as an organism, with each bee as a cell. By producing antimicrobial molecules and maintaining a stable microbiota, each bee contributes to the immunity of this superorganism (Simone-Finstrom, 2017).

As a result, almost all bee products contain antimicrobial molecules (Romanelli et al., 2011; Baracchi et al., 2012; Brudzynski and Sjaarda, 2015). Propolis is one of these, and its curative and antiseptic properties have been recognized since antiquity (Fokt et al., 2010). This complex mixture is composed mainly of plant resin and beeswax chewed together. Salivary compounds and, probably, other molecules are added during this chewing process. Bees use this product to fill holes in the hive and likely as a protective agent against wind and enemy access to the hive. The plant content of propolis makes its composition highly dependent on season and region. Nevertheless, Kujumgiev et al showed in 1999 that propolis from different regions all exhibited antibacterial and antifungal properties. Indeed, propolis helps colonies to fight against Gram + bacteria (Kujumgiev et al., 1999; Silici and Kutluca, 2005; Przybyłek and Karpiński, 2019), fungi and microsporidia such as *Vairimorpha spp* (Peng et al., 2012; Yemor et al., 2015; Arismendi et al., 2018; Mura et al., 2020; Naree et al., 2021) and parasites such as *Varroa destructor* and their associated viruses (Damiani et al., 2010; Drescher et al., 2017). Bees use propolis as a defense against infestations as well as for self-medication. Its consumption can indeed improve bee health and natural defenses against pathogens (Yemor et al., 2015; Drescher et al., 2017; Turcatto et al., 2018) and it is also known to have antioxidant properties (Russo et al., 2002; Kumazawa et al., 2004). In addition, propolis activates the bee immune system and generates high-level responses from associated genes to help bees fight microorganisms.

The properties of propolis and other hive products originate from the plant extracts that compose them (Cushnie and Lamb, 2006; Göçer and Gülçin, 2011; Peng et al., 2012; Meyuhas et al., 2015) as well as from the bees’ production of antimicrobial molecules. Antimicrobial peptides (AMPs) are one example of these molecules (reviewed by Danihlík et al., 2015). Their synthesis is induced by the presence of certain pathogens (Aronstein et al., 2010; Lourenço et al., 2013) and they are part of the bee humoral immune system. “Jelleins” are AMPs produced by workers and added to royal jelly for the queen. Most types of jelleins exhibit antimicrobial activity on bacteria and yeast, probably by acting on bacterial cell walls (Romanelli et al., 2011; Brudzynski and Sjaarda, 2015; Danihlík et al., 2015). They work synergistically with other AMPs, such as temporins, present in bee venom (Romanelli et al., 2011). Indeed, venom may also play a role as a protective agent against pathogens through its antimicrobial properties (Baracchi et al., 2012; Leandro et al., 2015; Wehbe et al., 2019). Some major venom compounds are found in the bee cuticle and in the wax (Baracchi et al., 2012), which suggests a more important role of venom in defense against pathogens. Beeswax also exhibits antimicrobial properties, summarized in the review by Fratini et al. (2016), as does bee bread (Bakour et al., 2019; Khalifa et al., 2020).

Honey possesses antimicrobial properties due to the presence of numerous antimicrobial compounds that act synergistically. Polyphenolic compounds from plants are the main antimicrobial agent in honey (Güneş et al., 2017) and explain how honeys with different origins exhibit different antimicrobial characteristics (Nolan et al., 2019). Bees use other plant products such as nectar, which they transform using specific enzymes including glucose oxidase. This enzyme catalyzes the glucose transformation and enables production of hydrogen peroxide, another potent antimicrobial in honey (Brudzynski et al., 2011; Nolan et al., 2019). Such enzymes play a key role in herd immunity, as they allow the production and dissemination of antimicrobials throughout the hive. Furthermore, bees tend to choose to consume the most antimicrobial honey when infected with the microsporidia *V. ceranae* (Gherman et al., 2014). This could be another example of self-medication. Honey also contains AMPs such as defensin-1, which contributes to its antimicrobial properties. The antimicrobial compounds in honey are very complex, due to variations in origins, compounds, and compound concentrations. They are well described in reviews by Kwakman and Zaat (2012) and Nolan et al. (2019). Finally, honey can also have antioxidant properties, as is the case with Manuka honey, which contains the highest rate of phenolic compounds known to reduce free radicals and oxidative damage (Stephens et al., 2010; Alvarez-Suarez et al., 2014).

To summarize, bees increase collective immunity by disseminating protective molecules throughout the hive in their products. These molecules include antimicrobial peptides (AMPs) produced directly by the bees and reactive molecules such as hydrogen peroxide (H_2_O_2_) produced indirectly by the catalysis of plant compounds by bee enzymes. Reactive molecules of bee products such as H_2_O_2_ could help degrade some pesticides, as demonstrated by several studies (Doong and Chang, 1998; Badellino et al., 2006). Bees also incorporate plant-derived antimicrobial molecules, such as polyphenols, into their products. Some components of propolis and honey such as p-coumaric acid can activate and regulate the immune system response and the process of detoxifying pesticides and other xenobiotics. These molecules and associated hive products act synergistically to address the most pathogens in the most effective way (Romanelli et al., 2011; Fratini et al., 2016). In addition, they show amazing potential for combating antibiotic resistance (Lerrer et al., 2007; Kwakman et al., 2011). Finally, an increasing number of studies are now looking at the specific microbiota of bees and the overall microbiota of the hive. Given that each bee has its own microbiota that can modulate the overall microbiota of the colony, this could be considered another kind of collective immunity of the bee. Since the individual and overall microbiota are also dependent on the external environment, the bacterial composition of the hive will also be discussed in the next paragraph.

### 4.3 External environmental factors helpful to bees

As seen in the previous paragraph, hive products (propolis and honey) contain many bioactive molecules, the majority of which come from plant extracts. Among flavonoids and phenolic acids (from wildflowers, fruit plants and aromatic plants), galantin and caffeic acid are known to have antimicrobial and antioxidant effects (Heijnen et al., 2001; Cushnie and Lamb, 2005; Cushnie and Lamb, 2006; Göçer and Gülçin, 2011; Meyuhas et al., 2015). In addition, p-coumaric acid, a ubiquitous component of pollen, activates the immune system and detoxification (Mao et al., 2013). This prepares the bee system for warding off potential future stressors, exemplifying how external molecules can help bees fight pathogen invasions. Unlike stressors, plants act as beneficial environmental factors. Interestingly, this phenomenon is fairly universal, since it does not depend on plant type or geographic area (Seidel et al., 2008; Damiani et al., 2010).

Bees also bring bacteria into the hive from their forays in their surroundings. These bacteria can be beneficial and integrate with the bacteria in the hive. Indeed, it appears that the bee microbiota is also dependent on the environment outside the hive (Jones et al., 2018). The presence of specific bacteria beneficial to bees can prevent the development of opportunistic bacteria by occupying ecological niches or by secreting antibacterial compounds (principle of competitive exclusion, Meszéna et al., 2006). In 2020, Wu and collaborators also showed that a healthy gut microbiota promotes endogenous detoxification in bees by upregulating the expression of P450-associated genes. Furthermore, the hive microbiota conditions the establishment of a beneficial microbiota on the larvae, promoting good development (Martinson et al., 2012; Powell et al., 2014). A healthy microbiota makes honeybees more resistant to many stressors (Koch and Schmid-Hempel, 2011; Steele et al., 2021) since an unstressed microbiota plays key functional roles for bees, such as pollen and carbohydrate digestion, molecule synthesis, immune system activation, and pathogen resistance, among others (Koch and Schmid-Hempel, 2011; Engel et al., 2012). Improving the health of the bee at the individual level makes the colony more resilient against all kinds of stressors. Finally, some bacteria also can degrade organic compounds such as pesticides (Zhang et al., 2014; Yuan et al., 2021) and thus reduce bee exposure to pesticides. Recently, in El Khoury et al. (2022), showed that the endogenous gut microbiota of honeybees has the ability to metabolize clothianidin, a compound highly toxic to bees. In fact, other factors such as UV and sunlight can degrade neonicotinoids (Gupta et al., 2008; Kurwadkar et al., 2016), as well as some reactive compounds such as reactive oxygen compounds or H_2_O_2_ (Doong and Chang, 1998; Badellino et al., 2006; Sablas et al., 2020). Thus, supplying additional beneficial bacteria (i.e., probiotics) could be a potential solution that beekeepers could implement to help honeybees resist to the multiple stressor exposure, as suggested by Chmiel et al. (2020).

## 5 Conclusion

**FIGURE 3:**
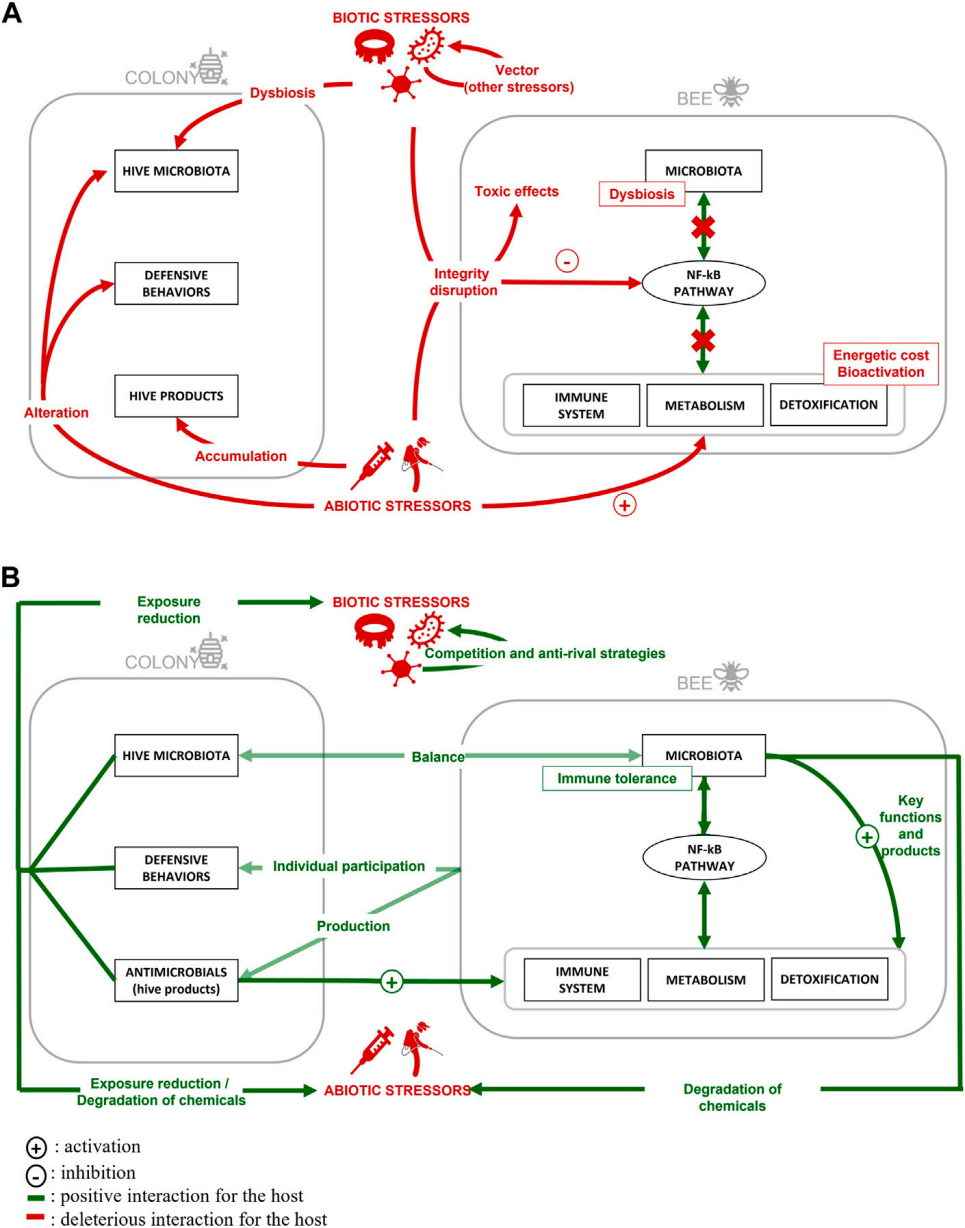
Effects of multiple stressor contamination on honey bees **(A)** and illustration of honey bee defense systems **(B)** at individual and colony levels.

In this review, we have described the interaction between stressors and the modes of action related to their synergic effects. We have also detailed the natural defenses of the honeybee. These interactions and their impacts on honey bees, as well as honey bee defense systems, are summarized in Figure 3. This valuable species not only provides us with hive products of great interest for agri-food and pharmaceutical fields, but it also pollinates our monocultures. However, honeybee health is declining, and some regions are now experiencing significant colony losses. Many studies have been carried out on this species to investigate the causes underlying this phenomenon. The species could also help to protect broader pollinator biodiversity, since *A. mellifera* can serve as a bioindicator of the state of the environment and has characteristics similar to those of other pollinators on an individual scale. It appears that honeybees suffer from exposure to multiple stresses in their environment, both biotic and abiotic, that generate a wide range of effects. These cumulative effects reinforce each other, multiply and engender additional impacts, since exposure to different stressors occurs simultaneously. Fundamental environmental factors such as temperature also interact with stressors, as do certain bee characteristics, such as bee caste. While it is extremely difficult to obtain a synthetic overview of the situation due to the number of parameters to be considered and individual differences among cases, certain modes of action related to stressor synergic effects have been identified. Synergistic stressors interactions (that negatively affect the bees) often result from a disruption of the immune response or detoxification that allows them to remain mismanaged in the body, or from an excessive defense response by the bee itself. Antagonistic interactions (that positively affect the bees) are most often a result of competition between stressors or activation of the bee’s own defenses.

The honeybee possesses a well-developed social immunity comprising complex behaviors and secretion/collection of various detoxification and antiseptic compounds that combat pathogens and reduce chemical toxicity. They also use their products to sterilize the hive and self-medicate, partly by incorporating external plant molecules. By consuming products like honey, they activate their defenses and improve their own health. External environmental factors can thus help bees to combat certain stressors through antimicrobial activity or by degrading anthropogenic pollutants such as pesticides. Beneficial factors combined with bee collective and social immunity should make them resistant to many stressors. This shows the seriousness of the situation for the health of bees and even more so for other pollinators that do not possess this type of protection. Nonetheless, the three types of defenses described here are encouraging and have great potential to function as strategies to help combat bee stressors. Genetic selection to promote hygienic behavior, allowing bees to retain some of their production, instead of feeding them sugar solutions during the winter, or placing hives in areas surrounded by plants with a high potential for detoxification activation represent solutions that are or could be considered in the future. Scientists also have a key role to play in safeguarding the honey bee. This literature review illustrates how fundamental it is to carry out integrative studies, targeting several stressors, both individually and in interaction, and using a holistic approach to bee health. The honey bee is also a superb biological model, enabling studies to be carried out on at different scales. This allows to study the effects and complex mechanisms of stressors in an accurate, targeted way (on a cage scale), and then to validate these effects and conclusions in the field (on an apiary scale). Various approaches can be used on bees to detect stressors. Targeted (e.g., enzyme-linked immunosorbent assay (ELISA), quantitative PCR, targeted mass spectrometry) or non-targeted analyses such as multi-omics approaches or microbiota analysis on honey bees should be seriously considered to detect not only the presence of stressors in the environment, but also to assess their bioavailability and toxicity. In conclusion, this review provides a sense of the magnitude of the problem, while the long-term chronic and behavioral effects of these stressors remain to be investigated further. Nevertheless, there is hope for the honeybee, as this knowledge can and should be used to develop concrete solutions to ensure their wellbeing.
